# Normal ranges of the fetal weight determined by ultrasound in the population of the Hospital das Clínicas of the Faculdade de Medicina da Universidade de São Paulo

**DOI:** 10.1016/j.clinsp.2025.100616

**Published:** 2025-03-15

**Authors:** Eliane Azeka Hase, Amanda Amaral, Stela Verzinhasse Peres, Rossana Pulcineli Vieira Francisco

**Affiliations:** Department of Obstetrics and Gynecology of the Hospital das Clínicas of the Faculdade de Medicina da Universidade de São Paulo, São Paulo, SP, Brazil

**Keywords:** Pregnancy, Gestational age, Ultrasound, Fetal Weight

## Abstract

**Objective:**

This study aimed to determine the normal range of fetal weight by ultrasound in pregnant women followed at the Obstetric Clinic of the Hospital das Clínicas of the Faculty of Medicine, University of São Paulo.

**Methods:**

This retrospective cohort study included singleton pregnant women without associated maternal diseases, at 15–41 weeks of gestation, who underwent their last ultrasound up to 7 days before birth. Fetal parameters analyzed for the normal range were biparietal diameter, femur length, head and abdominal circumference. 3rd, 10th, 50th, 90th, and 97th weight percentiles were determined for each gestational age. Newborns were classified by birth weight as Small (SGA), Appropriate (AGA), or Large (LGA) for gestational age.

**Results:**

Among 837 women admitted without maternal diseases, 136 were included and 379 examinations performed at 15–41 weeks of gestation. Multiple linear regression models were generated to develop the normal range of fetal weight. Three equations were selected, and six normal ranges were created considering the total population and stratified by fetal sex. Weight estimates were calculated for the 3rd, 10th, 50th, 90th, and 97th percentiles for each gestational age. Among the 136 newborns, 107 (78.7 %) were classified as AGA, 23 (16.9 %) as SGA, and 6 (4.4 %) as LGA.

**Conclusion:**

The normal range of the fetal weight determined by ultrasound in this population showed a good correlation with gestational age, enabling the fetal weight gain pattern evaluation. The equation based on four parameters, including days before birth, presented the lowest percentage error variation to estimate the normal range.

## Highlights

• Normal ranges of fetal weight as a function of gestational age was created.
• New parameter, days before birth, was incorporated for normal ranges of fetal weight.
• Parameter days before birth is useful for estimating fetal weight at scheduled birth.

## Introduction

Accurate Fetal Weight (FW) estimation directly impacts perinatal mortality and morbidity.^1^, ^2^, ^3^, ^4^ Fetal growth is influenced by various factors, including intrinsic growth potential,^2^ and specific characteristics of the population to which the pregnant woman belongs, such as socioeconomic, geographic, cultural, and environmental factors. Thus, identifying the growth profile of each population allows the correct diagnosis of growth disorders^1^,^5^, ^6^, ^7^ such as fetal growth restriction and macrosomia, improving maternal-fetal care and reducing public health costs. False-positive growth disorders lead to unnecessary testing to assess fetal well-being and growth, iatrogenic preterm birth, and increased maternal and family anxiety. This study aims to determine the normal range of FW by Ultrasound (US) in pregnant women followed up at this service to improve prenatal care and maternal and neonatal outcomes.

## Material and methods

This is a retrospective cohort study that enrolled pregnant women admitted to prenatal care at the Obstetric Clinic of the Hospital das Clínicas da Faculdade de Medicina da Universidade de São Paulo (HC-FMUSP), Brazil, Department of Obstetrics and Gynecology. The study was approved by the institution's Research Ethics Committee.

It was a non-probabilistic convenience sample. The inclusion criteria were pregnant women with an uncomplicated pregnancy, without associated maternal diseases, with a single fetus, at 15–41 weeks of Gestational Age (GA), undergoing at least one US examination with FW estimation at the HC-FMUSP, with last US up to 7 days before birth, and who gave birth at this service. The exclusion criteria were fetuses with malformations on obstetric US examination and Newborns (NBs) with structural malformations on neonatal clinical examination.

Fetal biometric parameters such as Biparietal Diameter (BPD), Head Circumference (HC), Abdominal Circumference (AC), and Femur Length (FL) were measured in millimeters (mm) on each US examination and were used to estimate FW.

The GA was expressed in weeks and based upon the date of the first day of the Last Menstrual Period (LMP) or on the US examination. The GA was based on LMP if it was confirmed by the first-trimester US estimated by the crown-rump length. After this period, the mean GA was estimated from fetal Biometric Parameters (BPD, HC, AC, and FL). If the LMP was uncertain or if the GA estimated by the LMP was different from the GA estimated by the US, the authors considered the GA estimated by the earliest US performed during pregnancy.

A medical team specialized in fetal medicine performed the US examinations at the HC-FMUSP Obstetric Clinic, using the Ecocce (Toshiba, Japan), Corevision SSA-350A (Toshiba, Japan), Tosbee (Toshiba, Japan), Voluson 730 Expert (USA), Voluson E8 (USA), Philips HDI (Netherlands), or Envisor (Philips, Netherlands) US machines and 3.5–5.0 MHz convex transducers. US data, including fetal biometric parameters and FW, were collected from the Computerized Report System (SILOG) of the HC-FMUSP Obstetric Clinic. Maternal, obstetric, and NB data were gathered from the computerized data system of the HC-FMUSP Obstetric Clinic (Microsoft Access 2007).

Postnatal fetal parameters were GA at birth (expressed in weeks and defined in prenatal follow-up), sex, and birth weight in grams (g). The NBs were classified by weight as Small (SGA), Adequate (AGA), or Large (LGA) for GA, according to the criteria adopted by the neonatology department of this service, based on the growth chart proposed by Fenton et al. (2013) .^8^ SGA was defined as weight lower than or equal to the 10th percentile for GA, AGA was defined as the weight between the 10th and 90th percentile, and LGA as weight equal to or above the 90th percentile.

The parameter days before birth used to create a normal range was defined as the period between the last US and birth.

### *Statistical analysis*

The data were descriptively analyzed using absolute and relative frequencies, measures of central tendency, and dispersion. To develop the normal range of FW for the study, multiple linear regression models were generated. These models considered the parameters BPD, AC, HC, FL, and days before birth as predictor parameters, and the Log10 of FW as outcome. Predictive parameters were tested separately and by interaction using the Backward, Forward, and Stepwise techniques. Among the seven equations generated, the authors selected the three with the highest coefficients of determination (R^2^ ≥0.90) and lowest percentage of FW prediction error using the formula:^7^ Error(%)=([estimatedweight−currentweight]/currentweight)×100.

The authors produced six normal FW ranges for the three equations, considering the total study population and the population stratified by sex. FW was estimated for each gestational week using the formula: $y=\beta 0\;+\;\beta 1*\mathrm{GA}\;+\;\beta 2*\mathrm{GA}\hat{2}$. The 3rd, 10th, 50th, 90th, and 97th percentiles of FW were estimated in the population using the equation: $P\;=\;10\hat{(}y(Z\left(\mathrm{Perc}\mathrm{enti}\mathrm{le}\right)\;*\;\left(\mathrm{Stan}\mathrm{dard}\;\mathrm{Error}\right)$.

Statistical significance was set at a 5 % descriptive level (*p* < 0.050). The data were entered into an Excel spreadsheet and analyzed using the SPSS software version 23.0 for Windows.

## Results

Among the 837 pregnant women without maternal diseases admitted to the service, 136 were included in the study to create the normal range of FW. These women underwent at least one US examination with FW estimation up to 7-days before birth and gave birth at this service.

The mean maternal age was 25.85 years (SD = 6.5), with a median of 26-years (13–43-years); most women were white (57.4 %); and 61 % had a previous childbirth. The mean number of pregnancies among the women analyzed was 2.4 (SD = 1.6), with a median of 2 (1–8). GA was based on LMP in 45.4 % of cases, and on the first US performed during pregnancy in 54.5 %. Among the study participants, 39 underwent US up to 20 weeks of pregnancy and only 3 (4.2 %) after 28-weeks.

A total of 379 US examinations were analyzed at 15–41 weeks of pregnancy (Table 1).

**Table 1:** 3rd, 10th, 50th, 90th, and 97th percentiles of the sample at 15 and 41 weeks (equations A, B, and C).

GA	n	P3			P10			P50			P90			P97		
		A	B	C	A	B	C	A	B	C	A	B	C	A	B	C
15	5	91.75	92.43	92.00	98.19	98.64	98.45	113.50	113.35	113.79	131.21	130.26	131.53	140.41	139.02	140.74
16	5	114.87	115.69	115.15	122.93	123.47	123.22	142.10	141.89	142.42	164.27	163.06	164.62	175.79	174.01	176.16
17	4	142.75	143.75	143.06	152.76	153.41	153.08	176.60	176.30	176.94	204.14	202.60	204.52	218.46	216.21	218.86
18	9	176.09	177.29	176.42	188.44	189.20	188.78	217.83	217.43	218.21	251.82	249.87	252.22	269.48	266.66	269.90
19	10	215.60	217.05	215.96	230.72	231.63	231.10	266.72	266.19	267.12	308.32	305.90	308.76	329.95	326.45	330.39
20	18	262.03	263.75	262.42	280.41	281.47	280.81	324.15	323.47	324.58	374.72	371.73	375.17	401.00	396.71	401.46
21	25	316.10	318.15	316.52	338.27	339.53	338.70	391.04	390.18	391.49	452.04	448.40	452.51	483.75	478.52	484.23
22	16	378.51	380.93	378.95	405.06	406.53	405.51	468.25	467.18	468.71	541.29	536.88	541.77	579.25	572.96	579.74
23	8	449.89	452.74	450.35	481.44	483.16	481.92	556.55	555.25	557.03	643.37	638.09	643.86	688.49	680.96	688.98
24	4	530.78	534.12	531.27	568.00	570.00	568.50	656.61	655.04	657.11	759.03	752.78	759.54	812.27	803.35	812.77
25	8	621.57	625.47	622.10	665.16	667.49	665.69	768.93	767.08	769.46	888.87	881.53	889.40	951.21	940.75	951.72
26	11	722.51	727.04	723.09	773.18	775.88	773.76	893.80	891.65	894.37	1033.23	1024.68	1033.78	1105.69	1093.53	1106.22
27	4	833.64	838.87	834.27	892.10	895.23	892.74	1031.26	1028.80	1031.89	1192.14	1182.29	1192.73	1275.75	1261.73	1276.32
28	7	954.74	960.76	955.45	1021.69	1025.31	1022.41	1181.07	1178.29	1181.78	1365.32	1354.09	1365.99	1461.07	1445.06	1461.71
29	13	1085.34	1092.24	1086.17	1161.46	1165.63	1162.29	1342.64	1339.54	1343.46	1552.09	1539.40	1552.87	1660.94	1642.83	1661.69
30	14	1224.69	1232.56	1225.67	1310.58	1315.37	1311.57	1515.02	1511.63	1516.00	1751.36	1737.16	1752.31	1874.19	1853.88	1875.11
31	19	1371.70	1380.64	1372.89	1467.90	1473.41	1469.10	1696.89	1693.24	1698.09	1961.60	1945.87	1962.78	2099.17	2076.61	2100.33
32	10	1525.00	1535.11	1526.45	1631.96	1638.25	1633.42	1886.54	1882.67	1888.03	2180.83	2163.57	2182.32	2333.78	2308.93	2335.25
33	18	1682.90	1694.26	1684.67	1800.93	1808.09	1802.73	2081.86	2077.86	2083.73	2406.62	2387.88	2408.52	2575.41	2548.31	2577.31
34	21	1843.41	1856.11	1845.58	1972.69	1980.82	1974.92	2280.42	2276.36	2282.76	2636.16	2615.99	2638.58	2821.04	2791.75	2823.49
35	22	2004.29	2018.43	2006.96	2144.85	2154.04	2147.61	2479.44	2475.43	2482.36	2866.22	2844.76	2869.29	3067.24	3035.89	3070.37
36	20	2163.09	2178.75	2166.36	2314.80	2325.13	2318.17	2675.89	2672.04	2679.51	3093.32	3070.71	3097.18	3310.27	3277.02	3314.23
37	33	2317.21	2334.45	2321.17	2479.73	2491.29	2483.84	2866.55	2862.99	2871.00	3313.72	3290.15	3318.51	3546.12	3511.20	3551.07
38	29	2463.96	2482.82	2468.71	2636.76	2649.63	2641.71	3048.08	3044.96	3053.49	3523.57	3499.26	3529.44	3770.69	3734.37	3776.79
39	21	2600.61	2621.14	2606.27	2783.00	2797.24	2788.91	3217.14	3214.59	3223.63	3719.00	3694.21	3726.10	3979.83	3942.41	3987.23
40	17	2724.55	2746.75	2731.20	2915.63	2931.29	2922.60	3370.46	3368.64	3378.16	3896.24	3871.25	3904.72	4169.49	4131.34	4178.36
41	8	2833.28	2857.14	2841.02	3031.99	3049.10	3040.12	3504.97	3504.03	3513.99	4051.73	4026.83	4061.73	4335.89	4297.38	4346.38

n, Number of cases; P, Percentile.

The three equations with the highest coefficients of determination (R^2^ ≥0.90) and lowest FW prediction variation error were A, B, and C and were used to estimate the FW of the population. Equation A was based on three parameters (AC, BPD, FL), and equations B and C were based on four (AC, BPD, FL, and days before birth), with R^2^ = 0.90 (error = 7.07 %), R^2^ = 0.91 (error = 6.84 %), and R^2^ = 0.91 (Error = 6.83 %), respectively (Table 2).

**Table 2:** Linear regression models to create the normal FW range for the present sample.

Equations (data entry technique)	Parameters	Linear regression = database (*n* = 136)	R^2^	Error (%)
A (Stepwise)	AC, BPD, and FL	Weight_Log10_ = 0.741618390842443+ (−0.0000592460422010622* (BPD*AC)) + (0.00106319494748685*FL) + (0.00735689752184094*AC) + (0.022304256488123*BPD)	0.90	7.07
B (Forward)	AC, BPD, FL, and days before birth	Weight_Log10_ = 0.771246022461875 + (−0.0000571937414961564*(BPD*AC)) + (0.00135846909462373*FL) + (0.00717485097466084*AC) + (0.0216154673171614*BPD) + (0.00346941874495245*days before birth)	0.91	6.84
C (Backward)	AC, BPD, FL, and days before birth	Weight_Log10_ = 0.782905034761583 + (0.0214552864486131*BPD) + (0.00743501892929378*AC) + (0.0000158779135163121*(BPD*FL)) + (−0.0000601560133000023*(BPD*AC) + (0.00345057763744124*days before birth)	0.90	6.83

AC, Abdominal Circumference; BPD, Biparietal Diameter; FL, Femur Length.

**Fig. 1:**
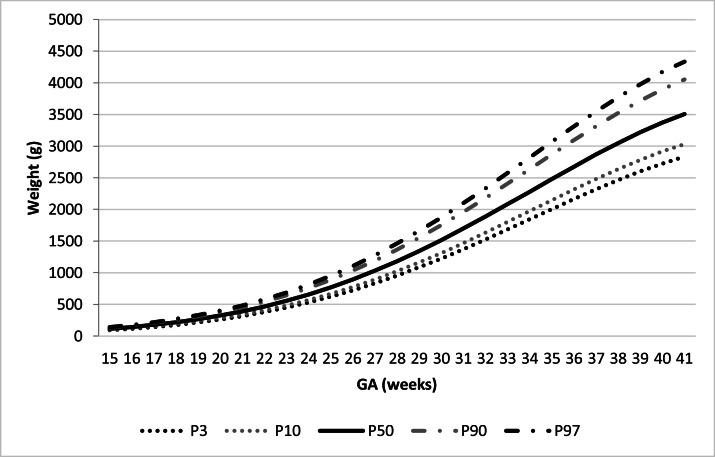
Curve C (Backward): FW as a function of GA (weeks) with the 3rd, 10th, 50th, 90th, and 97th Percentiles (P).

**Fig. 2:**
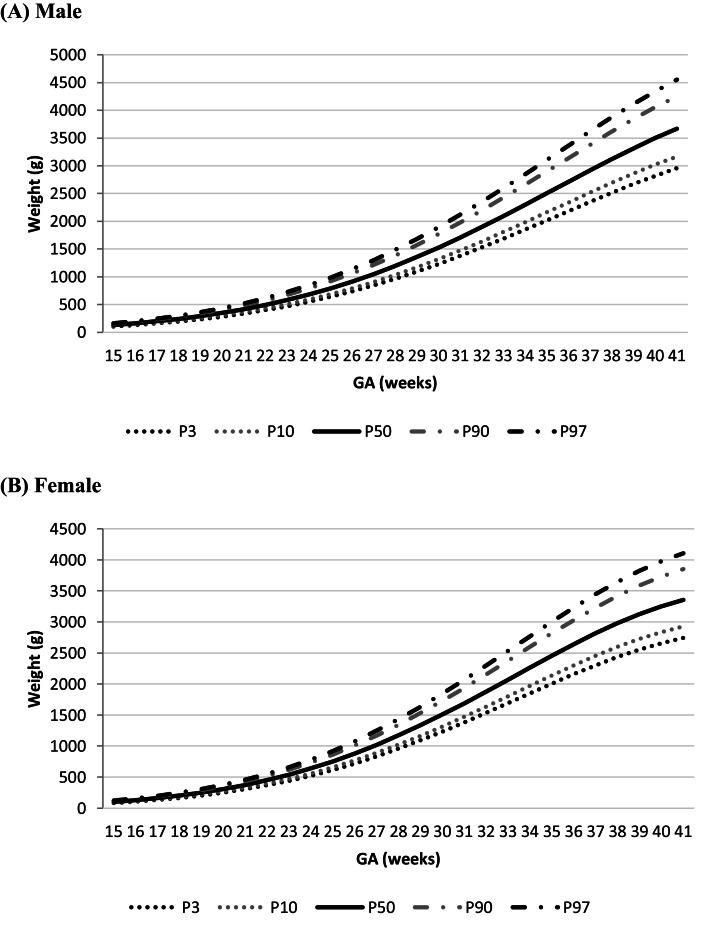
Curve C (Backward): FW as a function of GA (weeks) with the 3rd, 10th, 50th, 90th, and 97th Percentiles (P) by sex.

The authors created six normal FW ranges for the three equations, considering the total study population and the population stratified by sex. FW was estimated for the 3rd, 10th, 50th, 90th, and 97th percentiles for each GA (Table 1). Equation C presented the lowest error in the FW prediction variation. Figs. 1 and 2 represent the FW range as a function of GA for the total sample and stratified by sex.

The comparison of the 50th percentile FW between sexes over the GAs exhibited that male fetuses had a mean increase of β = 0.84 (CI95% 0.77 - 0.91) grams compared to females (p < 0.001).

The last US was performed on a mean of 3 days (SD = 2.3) before birth, ranging between 0 and 7 days, at a mean GA of 37.0 weeks (SD = 3.5). The mean FW was 2773.79*g* (SD = 752.89), ranging between 337*g* and 4202*g*.

The mean GA at birth was 37.50 weeks (SD = 3.4), and the mean weight of the NBs was 2888.51*g* (SD = 738.12) (Table 3).

**Table 3:** Gestational age (GA) on last US (weeks), GA at birth (weeks), FW on last US (g), NB weight (g) and US days before birth represented as mean, median, SD, and minimum and maximum values.

	GA on last US	GA at birth	FW on last US	NB weight	Days before birth
**N**	136	136	136	136	136
**Mean**	37.01	37.50	2773.79	2888.51	3.07
**SD**	3.52	3.45	752,90	738,12	2,33
**Median**	37.57	38.00	2751.50	2960.00	3.00
**Minimum**	20.28	22.57	337	590	0
**Maximum**	41.86	42.00	4202	4440	7

N, Number of cases.

Among the 136 NBs, 71 (52.27 %) were male and 65 (47.8 %) were female. At birth, 107 (78.7 %) were classified as AGA, 23 (16.9 %) as SGA, and 6 (4.4 %) as LGA, according to the normal range for NBs,^8^ with no significant difference between them (Table 4).

**Table 4:** NB distribution as AGA, SGA, and LGA by sex.

			Fetal sex		Total
			Male	Female	
**Weight classification**	AGA	N	57	50	107
		% within fetal sex	80.3 %	76.9 %	78.7 %
	SGA	N	11	12	23
		% within fetal sex	15.5 %	18.5 %	16.9 %
	LGA	N	3	3	6
		% within fetal sex	4.2 %	4.6 %	4.4 %
**Total**		N	71	65	136
		% within fetal sex	100.0 %	100.0 %	100.0 %

Note: Fisher's Exact test, *p* = 0.890.

## Discussion

Most low-risk pregnant women seeking the service were young, white, and with a previous birth. Since this study was conducted in a tertiary public hospital, the population of low-risk pregnant women was smaller, which limited the number of cases included in the sample. The authors created six normal ranges of FW determined by the US as a function of GA for the total sample and stratified by sex.

Accurate FW estimation is fundamental in prenatal follow-up^1^, ^2^, ^3^ because it helps identify fetal growth disorders associated with several maternal and fetal factors. These disorders may be related to the genetic constitution of the fetus, epigenetics, or adverse factors such as maternal diseases, smoking, alcohol consumption, nutritional status, medication use, and genetic or chromosomal disorders, among other causes associated with increased fetal and neonatal morbidity and mortality.^1^^,^^3^,^7^ Numerous studies have used the US to estimate a more accurate FW.

Most services and comparative studies use the range of FW determined by the US proposed by Hadlock et al.^7^ to evaluate fetal growth. Although this range has a good correlation with birth weight, the percentile value corresponding to a given estimated weight can differ according to the growth range used. Hence, in the specific service, it may not reflect the actual percentile of the population. Each service should develop its own FW range at different GAs to determine the growth pattern of its specific population.^7^ This would improve the diagnosis of growth disorders. Thus, the authors developed this study to create a normal growth range for the population by estimating the FW of each percentile for a given GA.

The range proposed by Hadlock et al.^7^ is calculated with a mathematical equation using four fetal biometric parameters (BPD, HC, AC, and FL). The authors use this range as the standard reference in this service, with a percentage error of 8.0 %. The percentage error found in this study was lower, ranging between 6.83 % and 7.07 %. The analysis of the parameters included in the equations exhibited that HC was absent in the three equations selected. This may be explained because the measurements considered in the study were obtained up to seven days before birth in fetuses with a mean GA superior to 37 weeks. At advanced GAs, especially near term, the size and position of the fetal head in the uterus can hinder the identification of head boundaries for measuring HC, thereby reducing the accuracy of this measurement.

J. Stirnemann et al.^9^ proposed an international weight range. They utilized the same methodology in eight countries, starting at 22 weeks of GA. The estimated FW percentiles were similar to those of NBs at the end of the third trimester. However, premature fetuses exhibited significant differences in the percentiles of estimated weights between fetuses and NBs. They compared the weight of fetuses and NBs, considering the last US performed up to 14 days before birth, with a mean of 7.7 days (0–14 days), and 1.4 % performed on the day of birth, contrasting this study, which assessed fetuses up to 7 days before birth. In Brazil, Fujita et al.^10^ conducted a retrospective study using the formula proposed by Hadlock et al.,^7^ which associated maternal and fetal data to estimate FW, including maternal height, pre-gestational body mass index, fetal sex, and GA. That study diverges from the present proposal, as the authors intended to develop a specific normal FW range for this service.

This retrospective study analyzed previously collected data. Thus, some clinical data that may interfere with FW were not evaluated because were not described in the patients’ medical records, such as eating habits, smoking, and body mass index, limiting the present study.

The authors created six normal ranges of FW as a function of GA for the total study population and the population stratified by sex. Among the three equations obtained, equation C (Backward) presented the lowest percentage variation error (6.83 %). This equation included days before birth. This is one of the strengths of the present study. The authors created FW ranges with a lower percentage variation error than the range proposed by Hadlock,^7^ which is currently used. The authors also incorporated a new parameter, days before birth, which is not considered in any of the equations proposed in the literature. Although the FW ranges determined by the US demonstrated a good correlation with GA, considering the percentage variation error, the authors suggest using equation C for estimating FW in the studied population. However, when using the parameter days before birth to estimate FW on the day the US is performed, this parameter should be set to zero in the proposed formula, as this will nullify the parameter in the formula and estimate FW on the day of the US. This parameter is also useful for estimating FW at scheduled birth on days following the date of the current US, helping to estimate FW up to 7 days before the scheduled birth.

Some studies identified different FW between males and females, revealing that male fetuses tend to be heavier than female fetuses.^5^^,^^10^^,^^11^ Based on this, the authors used equation C to compare the sexes and to evaluate FW at the 50th percentile at different GAs. The present sample exhibited higher mean weight gain in male than in female fetuses.

The analysis of postnatal parameters revealed that the last US was performed close to birth, with a mean interval of 3 days, and that the mean GA at the last US was similar to the mean GA of NBs. This consistency can be attributed to the low-risk profile of the pregnant women analyzed; as they had no associated maternal diseases, most NBs were born at full term and classified AGA.

Among the 136 NBs, the sex distribution was similar both across the entire sample and within each birth classification group. This indicates that there was no significant difference between groups regarding the sex of the fetuses, facilitating more accurate comparisons of the findings within this sample.

Therefore, based on the results obtained and the fetal growth profile of the population, the authors aim to enhance maternal-fetal care by identifying fetal growth disorders. By achieving this, the authors anticipate lowering public health costs and reducing anxiety levels for mothers and their families.

### *Perspectives*

This study lays the groundwork for improving and developing additional fetal growth ranges, considering maternal and fetal factors that might influence fetal development in this population. By acquiring a broader spectrum of cases, including high-risk pregnancies, and developing a prospective study with a larger sample, the authors will be able to evaluate the applicability of the ranges to these cases. Furthermore, the authors can undertake comparative analyses with other international and national standard growth ranges.

## Conclusion

The normal range of fetal weight determined by ultrasound in this population showed a good correlation with gestational age, enabling the fetal weight gain pattern evaluation. The equation based on four parameters, including days before birth, presented the lowest percentage error variation to estimate the normal range.

## Ethics statement

The studies involving human participants were reviewed and approved by the institution's Research Ethics Committee (Comissão de Ética para Análise de Projetos de Pesquisa do HCFMUSP).

## Funding

This research did not receive any specific grant from funding agencies in the public, commercial, or not-for-profit sectors.

## Conflicts of interest

The authors declare no conflicts of interest.
